# N-glycomic Complexity in Anatomical Simplicity: *Caenorhabditis elegans* as a Non-model Nematode?

**DOI:** 10.3389/fmolb.2019.00009

**Published:** 2019-03-12

**Authors:** Katharina Paschinger, Shi Yan, Iain B. H. Wilson

**Affiliations:** ^1^Department für Chemie, Universität für Bodenkultur, Wien, Austria; ^2^Institut für Parasitologie, Veterinärmedizinische Universität, Wien, Austria

**Keywords:** glycome, nematode, N-glycan, *Caenorhabditis*, glycosyltransferases

## Abstract

*Caenorhabditis elegans* is a genetically well-studied model nematode or “worm”; however, its N-glycomic complexity is actually baffling and still not completely unraveled. Some features of its N-glycans are, to date, unique and include bisecting galactose and up to five fucose residues associated with the asparagine-linked Man_2−3_GlcNAc_2_ core; the substitutions include galactosylation of fucose, fucosylation of galactose and methylation of mannose or fucose residues as well as phosphorylcholine on antennal (non-reducing) *N-*acetylglucosamine. Only some of these modifications are shared with various other nematodes, while others have yet to be detected in any other species. Thus, *C. elegans* can be used as a model for some aspects of N-glycan function, but its glycome is far from identical to those of other organisms and is actually far from simple. Possibly the challenges of its native environment, which differ from those of parasitic or necromenic species, led to an anatomically simple worm possessing a complex glycome.

## Introduction

*Caenorhabditis elegans* is one of the top non-mammalian eukaryotic model organisms and is widely used in developmental and aging studies (Corsi et al., 2015; Maglioni et al., 2016). Furthermore, as a nematode, it is genetically related to a number of parasites of humans, other animals and plants (Blaxter, 2011), which impact human health and agricultural productivity. However, *C. elegans* itself is not a parasite and, at least in the laboratory, is primarily hermaphrodite, unlike the majority of nematodes which are dioecious (i.e., have separate male and female sexes); it is a relatively simple anatomically bacterivore, consisting of an intestine surrounded by muscle, nerve and reproductive tissue, with a defined number of nuclei and a rapid lifespan. Its genome was the first to be sequenced of any multicellular organism (The C elegans Sequencing Consortium, 1998) and a wide range of mutant strains as well as RNAi clones are available; it is also amenable to engineering via the CRISPR/Cas9 system (Farboud, 2017). All these factors facilitate the investigation of gene function in *C. elegans*.

As any organism, it can be assumed that all its cell surfaces are covered in glycoconjugates; there have been various reports on its N-, O- and lipid-linked glycans over the past two decades and much new knowledge has been gained since our last review on *C. elegans* glycosylation written in 2008 (Paschinger et al., 2008). What is remarkable is that a consensus as to the actual structures was slow to emerge (Haslam and Dell, 2003) and we are still discovering new glycan variants; also, of the enzymes necessary to process and synthesize its glycome, only the activity of a few glycosyltransferases and glycosidases has been characterized (see below), while altered lectin binding or toxicity has been observed for a few “glycomutant” strains (Butschi et al., 2010; Schubert et al., 2012). Thus, in contrast to the well-defined and tractable genome, we still do not understand the glycome and how it is synthesized. What is certain is that unusual glycans do occur in *C. elegans*, some of which are also found in other nematodes, and that there are lectins which recognize certain glycan motifs.

## N- Glycosylation

The biosynthesis in the endoplasmic reticulum of the dolichol-linked Glc_3_Man_9_GlcNAc_2_ tetradecasaccharide precursor is expected to occur in the same way as for most eukaryotes. Primarily we can rely on the homologies to known *alg* (asparagine-linked glycosylation) genes, encoding various *N-*acetylglucosaminyl-, mannosyl- and glucosyltransferases as well as the detection of the full tetradecasaccharide in protein-linked form when the first processing glycosidase (glucosidase I encoded by the *agl-1* gene) is knocked-down (Struwe et al., 2009; Katoh et al., 2013; Akiyoshi et al., 2015). As with most eukaryotes, the Glc_1_Man_9_GlcNAc_2_ is the probable key intermediate in a cycle involving glucosidase II, calnexin and a glucosyltransferase in quality control of glycoprotein folding in the endoplasmic reticulum (Buzzi et al., 2011; Bai et al., 2018).

More species-specific, however, is what happens in the Golgi apparatus, after removal of three α1,2/3-linked glucose (Katoh et al., 2013) and four α1,2-linked mannose residues (Wilson, 2012), to the key intermediate Man_5_GlcNAc_2_. In wild-type worms, the major portion of this structure is modified by *N-*acetylglucosaminyltransferase I (GlcNAc-TI; designated *MGAT1* in many species), which in *C. elegans is* encoded by three different genes (*gly-12, gly-13*, and *gly-14*) which must all be knocked out in order to abolish GlcNAc-TI activity, resulting in an accumulation of Man_5_GlcNAc_2_ as the major component of the N-glycome (Zhu et al., 2004); recently we have additionally found a number of unusual glycans with galactose and/or fucose residues in the relevant triple knock-out (Yan et al., 2018b). Despite the large shift in the glycome, *gly-12*;*gly-13;gly-14* mutant worms survive quite happily under laboratory conditions, but have a different sensitivity to bacteria as compared to the wild-type (Shi et al., 2006).

**Figure 1:**
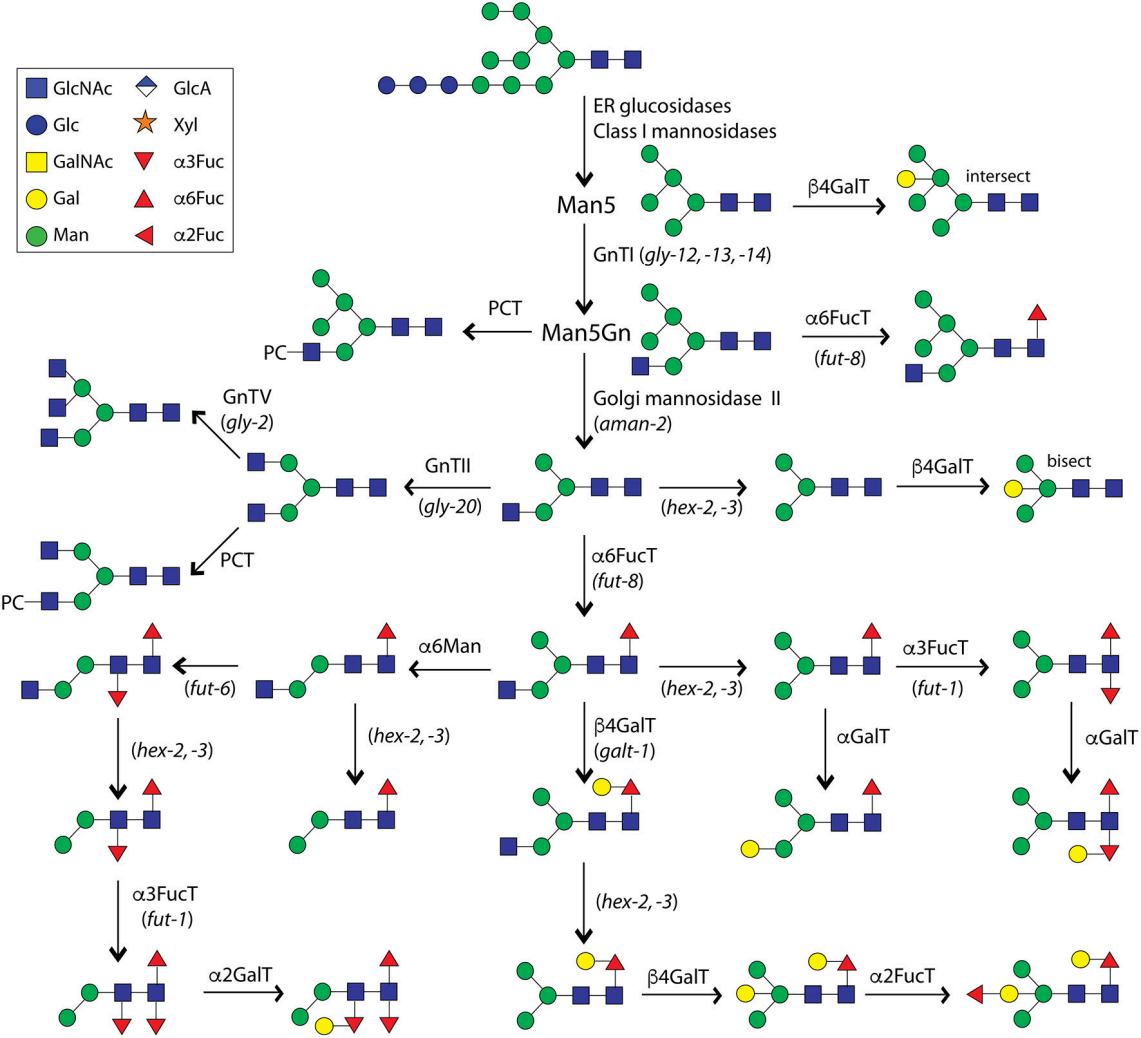
Processing of N-glycans in *C. elegans*. This summary, which is an updated version of our 2008 figure (Paschinger et al., 2008), is not intended to be exhaustive, but shows a number of reactions proven *in vitro* or postulated to exist *in vivo*. Abbreviations for proven or potential enzymes are shown above/beside the arrows (FucT, fucosyltransferase; GalT, galactosyltransferase; GnT, *N-*acetylglucosaminyltransferase; Hex, hexosaminidase; Man, mannosidase; PCT, phosphorylcholinyltransferase), whereas names of genes (in italics) relevant for certain biosynthetic steps are indicated in brackets below/beside the arrows. The Symbolic Nomenclature for Glycans is used throughout this article (e.g., blue squares for GlcNAc, green circles for Man, yellow circles for Gal, and red triangles for Fuc residues; see also the top left panel).

The product of GlcNAc-TI, Man_5_GlcNAc_3_, is the substrate especially for Golgi α-mannosidase II, which is conserved in multicellular eukaryotes; the activity of this enzyme has been determined for the *C. elegans* homolog as well as determining a large shift in the N-glycome in the relevant *aman-2* mutant strain (Paschinger et al., 2006). The product of this enzyme is a Man_3_GlcNAc_3_ structure (MGn in the Schachter nomenclature), which is the presumed substrate for the next two ‘branching’ *N-*acetylglucosaminyltransferases (GlcNAc-TII and GlcNAc-TV; GLY-20 and GLY-2, corresponding to mammalian *MGAT2* and *MGAT5*) as well as Golgi β-hexosaminidases HEX-2 and HEX-3 (Chen et al., 2002; Warren et al., 2002b; Zhang et al., 2003; Gutternigg et al., 2007). Unlike filarial nematodes, there is no GlcNAc-TIV homolog in *C. elegans* and thus no tetra-antennary N-glycans. However, these early processing events (see Figure 1) to generate either branched N-glycans or the simplest paucimannosidic forms are relatively unspectacular as these or similar reactions occur in a range of other invertebrates including insects.

## Maximal Degree of N-Glycan Diversity

**Figure 2:**
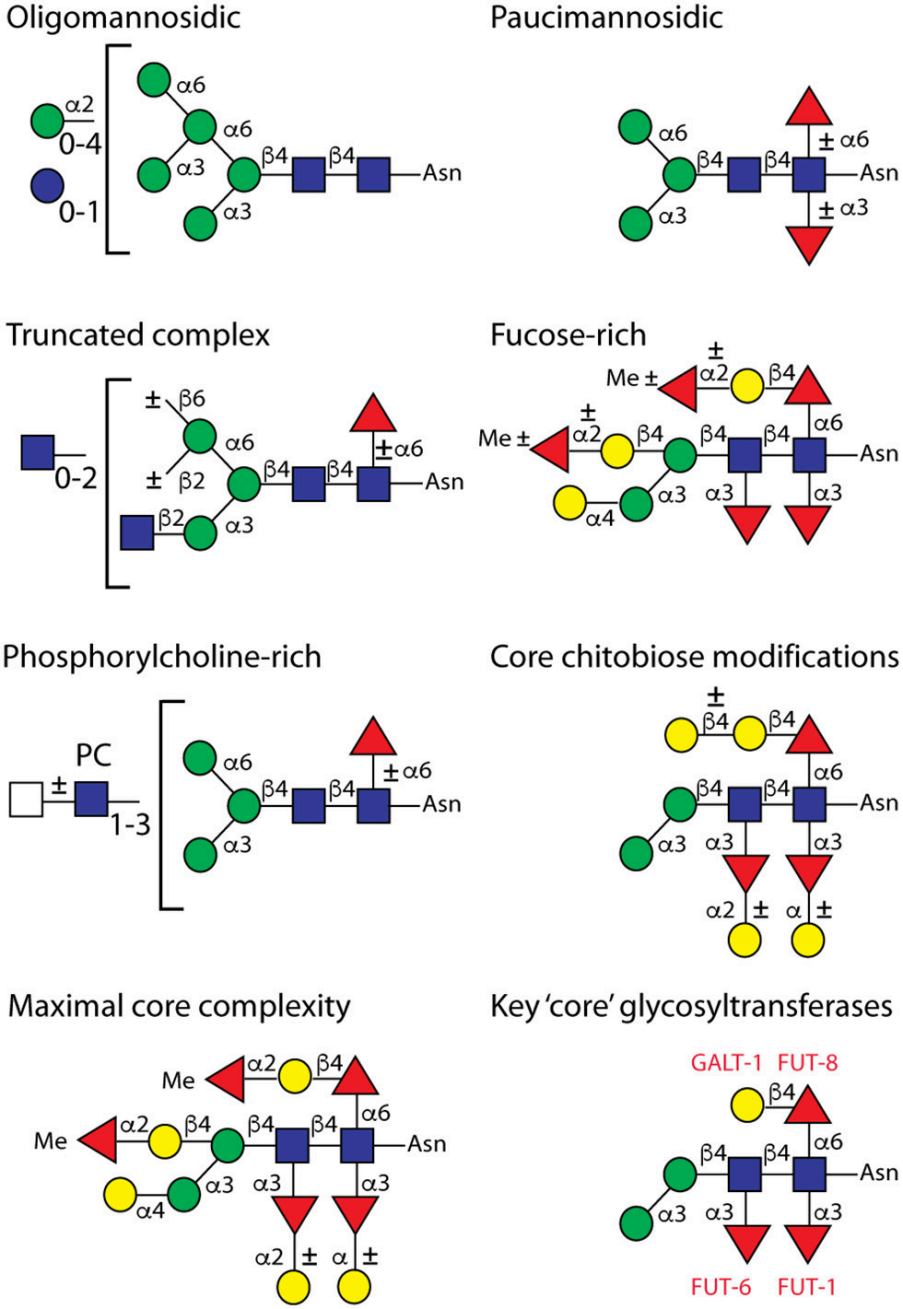
Example *C. elegans* wild-type N-glycan structures. This summary is an updated version of our 2008 figure (Paschinger et al., 2008), which was originally based on the classification of Haslam and Dell (2003). The classifications are not rigid, as “fucose-rich” and “core chitobiose modified” glycans are also paucimannosidic in the sense that they contain two mannose residues. The maximal core complexity shows a glycan found upon hydrazine release and the definition of the enzymes (red lettering) modifying the core is based on *in vitro* assays and glycomic analyses of mutants.

Various potential structures for highly fucosylated N-glycans of *C. elegans* have been postulated over the years and, in addition to modifying the reducing-terminal (proximal) GlcNAc to yield core difucosylated glycans of the type found in many invertebrates, the presence of fucose on the second (i.e., distal) core GlcNAc was without doubt; also galactosylation of the three core fucose residues was observed, although the exact linkages of galactose to the α1,3-fucoses remained obscure (Hanneman et al., 2006). However, often a fourth fucose on a mannose residue or distal fucosylation on a trimannosyl-containing structure have been suggested, but both such annotations can now said to be incorrect. Indeed, a big surprise to us was discovering bisecting galactose on *C. elegans* N-glycans (i.e., modification of C4 of the core β1,4-mannose as verified by enzymatic digestion, ESI-MS/MS and NMR; see Figure 2), which in turn could be α1,2-fucosylated (Yan et al., 2015a). Typically, bisecting residues are β1,4-linked GlcNAc as in mammalian and slime mold glycans (Hykollari et al., 2013), but α-linked GlcNAc or a galactofuranose have been found in fungal species (Buser et al., 2010; Hykollari et al., 2016). Furthermore, we detected intersecting galactose on the N-glycans of the triple GlcNAc-TI knockout or α-galactose on the α1,3-mannose in a number of mutant worm strains (Yan et al., 2015b, 2018a,b). When using hydrazine or the recombinant PNGase Ar, we could determine that the proximal core α1,3-fucose is modified with an α-linked galactose (Yan et al., 2018a), rather than β-linked as previously concluded; a fifth position for α1,2-fucosylation on wild-type or mutant N-glycans is on the proximal Gal β1,4Fuc α1,6 (“GalFuc”) moiety (Yan et al., 2015b, 2018a). Methylation of either α1,2-fucose (on the bisecting Gal or on the GalFuc) or mannose is a signature of some “mature” glycan structures (see Figure 2). While glycan methylation is reduced in a strain with a deficient candidate *S-*adenosylmethionine transporter gene (Wohlschlager et al., 2014), variations or reductions in N-glycan fucosylation have been noted in mutants with ablated GDP-fucose metabolism or defective Golgi trafficking (Barrows et al., 2007; Struwe and Reinhold, 2012).

**Figure 3:**
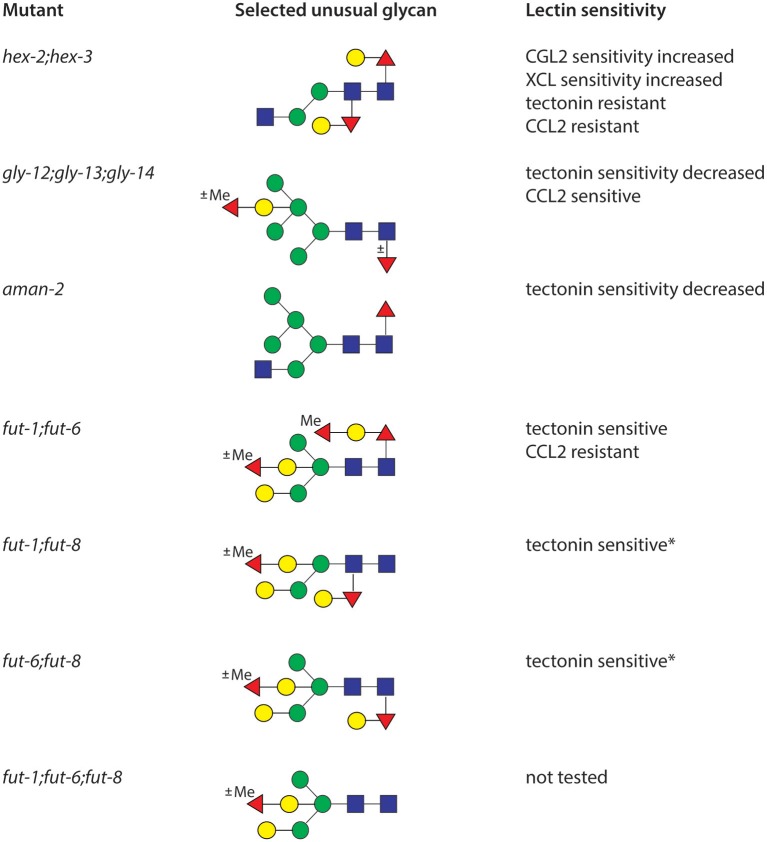
Correlations of N-glycan structure and lectin sensitivity. Example glycans from double and triple mutants analyzed in our laboratory are shown as well as a summary of data from the Aebi/Künzler group on their sensitivity to nematoxic carbohydrate binding proteins isolated from fungi; CCL2 and CGL2 from *Coprinopsis* respectively bind core α1,3-fucose and GalFuc, tectonin from *Laccaria bicolor* to methylated sugars and XCL from *Xerocomus chrysenteron* to GlcNAc. Note that also various single mutants (*bre-1, ger-1, galt-1, fut-1, fut-6, fut-8, gly-12, gly-13, gly-14*, and *gly-20*) are also displaying sensitivity or resistance toward *Aleuria aurantia* lectin (AAL), CGL2 and CCL2, while it is assumed that all double mutants with a defective *fut-8* gene (marked with an asterisk, ^*^) are also CGL2 resistant like the single mutant.

The enzymatic basis for these elaborations is only partly understood. Specifically, we identified the relevant α1,3- and α1,6-fucosyltransferases (FUT-1, FUT-6, and FUT-8) for modifying the proximal and distal core GlcNAc residues (Paschinger et al., 2004, 2005; Nguyen et al., 2007; Yan et al., 2013) and have proven their activity by *in vitro* tests and analysing the impact on the N-glycome. While FUT-8 accepts substrates with a non-reducing terminal GlcNAc, FUT-1 prefers a Man_3_GlcNAc_2_ structure and FUT-6 does not act when the α1,6-mannose is present (see Figure 1); however, FUT-6 can also act as a Lewis-type enzyme *in vitro*. Thereby, FUT-1 and FUT-6 have a rather unusual substrate specificity, e.g., in contrast to proximal core α1,3-fucosyltransferases in plants and other invertebrates, FUT-1 is not dependent on the prior action of GlcNAc-TI. Despite have major changes in the N-glycome (see Figure 3), single, double and triple fucosyltransferase knock-outs as well as the *hex-2;hex-*3 double mutant are perfectly viable in the laboratory; in the case of the *fut-1*;*fut-6*;*fut-8* mutant, significant amounts of fucose are present on the bisecting galactose rather than on the core, suggesting a certain biological and biosynthetic flexibility in the worm (Yan et al., 2015a). Some of these mutants display either resistance or increased sensitivity to fungal nematoxic carbohydrate binding proteins such as CCL2, CGL2, or tectonin (Schubert et al., 2012; Yan et al., 2012; Wohlschlager et al., 2014). “N-glycomutants” have been very valuable in tracking down modifications otherwise present on a number of low abundance structures, but it is unknown as to how they would survive biological stresses in a “wild” setting.

The substrate specificity has also been defined for GALT-1 which β-galactosylates the α1,6-fucose and generates an epitope recognized by a nematoxic lectin (Titz et al., 2009; Butschi et al., 2010). However, neither the nature of the bisecting galactosyltransferase, the α-galactosyltransferases, the α1,2-fucosyltransferases and methyltransferases are known nor whether either of the two α1,2-fucosyltransferases characterized from *C. elegans* (Zheng et al., 2002, 2008), out of some 20 homologs, have any role in N-glycan modification. Additionally, yet to be characterized are large numbers of CAZy GT14, GT31, and GT92 family members (Lombard et al., 2014) related to various UDP-sugar utilizing glycosyltransferases.

Other than the complex core regions, there are many studies indicating the presence of phosphorylcholine on the N-glycans. Certain is the occurrence of this zwitterionic modification on the antennae of some *C. elegans* N-glycans (Haslam et al., 2002; Cipollo et al., 2005; Hanneman et al., 2006; Paschinger et al., 2006; Yan et al., 2012, 2015a). Whereas a phosphorylcholinyltransferase activity in worm extracts was reported (Cipollo et al., 2004b), we can still only guess as to whether the relevant enzyme is encoded by homologs of bacterial genes involved in transfer of PC to lipopolysaccharides. However, transfer of this moiety to nematode N-glycans is seemingly dependent on the prior action of GlcNAc-TI (Houston et al., 2008). The maximal length of the PC-modified antennae is still to be determined and, in contrast to many invertebrates, anionic substitutions such as glucuronic acid or sulfate have not been detected on the N-glycans of *C. elegans*.

## O-Glycosylation

**Figure 4:**
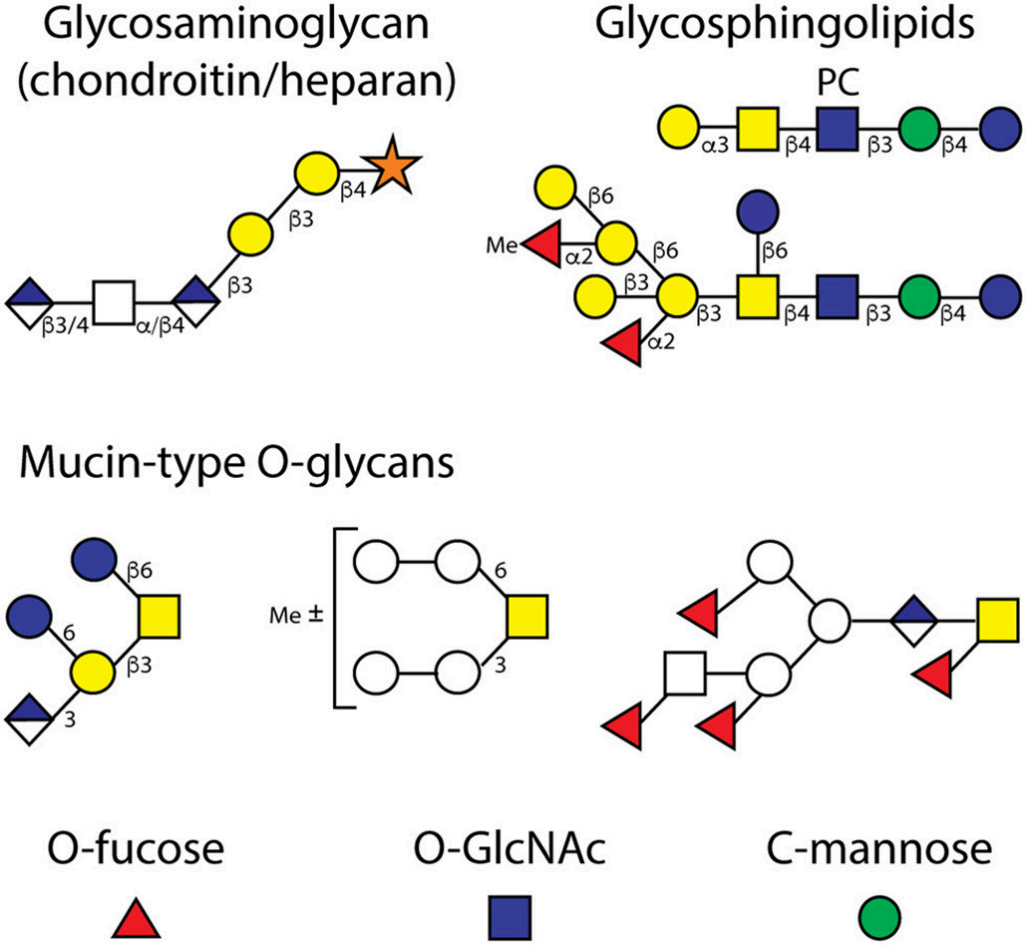
Examples of O-glycans and glycolipids. Depicted are example structures of (i) O-linked glycosaminoglycans (the α/β4 and β3/4 indicate the difference between chondroitin and heparan chains), (ii) neutral and zwitterionic glycosphingolipids, (iii) “core I” and “core II” mucin-type O-glycans, whereby the latter with a GalNAc modified with fucose and glucuronic acid are unrelated to the core 2 structures found in mammals, and (iv) the simple monosaccharide modifications of EGF, TSR and cytosolic proteins (O-Fuc, O-GlcNAc, and C-Man). The glycolipid and O-glycan structures are based on the work of various laboratories (Gerdt et al., 1997, 1999; Guérardel et al., 2001; Cipollo et al., 2004a; Griffitts et al., 2005; Palaima et al., 2010; Parsons et al., 2014; Wohlschlager et al., 2014).

The two major forms of O-glycans in *C. elegans* are the mucin-type GalNAc-Ser/Thr-based and glycosaminoglycan-type Xyl-Ser-based forms. As in mammals, there is a family of peptide-modifying GalNAc transferases (Hagen and Nehrke, 1998), thereafter the action of a galactosyltransferase yields the typical “core 1” Gal β1,3GalNAc mucin-type disaccharide (Ju et al., 2006); there is apparently no “core 2” GlcNAc modification as in mammals, but rather glucose in β1,6-linkage to the GalNAc (Guérardel et al., 2001), for which there is a cognate glucosyltransferase (Warren et al., 2002a). Terminal and internal glucuronic acid as well as terminal fucose residues are also found, partly in the context of non-standard core structures (see Figure 4), and changes in the expression of anionic, fucosylated or methylated O-glycans have been noted in different *srf*, *bus* and *samt-1* strains with reduced bacterial adhesion or altered lectin/tectonin binding (Cipollo et al., 2004a; Palaima et al., 2010; Parsons et al., 2014; Wohlschlager et al., 2014); thus, bacteria can target the wild-type mucins in order to adhere to the *C. elegans* cuticle. Indeed, it is also known from humans that the natural glycan structures do offer “points of entry” for pathogens; nevertheless, these glycans were not eliminated during evolution and so suggests they have other physiological roles.

The formation of chondroitin and heparan chains (see Figure 4), on the other hand, is initiated by the peptide *O*-xylosyltransferase, which is encoded by the *sqv-6* gene (Hwang et al., 2003a; Brunner et al., 2006), of which there is only one isoform (rather than two as in vertebrates). A range of enzymes synthesize the extension on the serine-linked xylose residue and defects in the relevant *sqv* or *rib* genes have revealed a number of important developmental roles for glycosaminoglycans in *C. elegans* (Hwang et al., 2003b; Franks et al., 2006); RNAi phenotypes verify the importance of this pathway (Akiyoshi et al., 2015). Analytical studies on these chains are primarily based on analyses of disaccharides (Toyoda et al., 2000), but we have recently observed longer chains with up to 25 monosaccharide units when using hydrazinolysis of worm glycopeptides, but not the same extra galactose or phosphorylcholine residues on “GAG-like” oligosaccharides as in the parasite *Oesophagostomum dentatum* (Vanbeselaere et al., 2018). In *C. elegans*, both 4-sulphation of chondroitin and 2- and 6-sulphation of heparan are at relatively low levels as compared to the total glycosaminoglycan content (Izumikawa et al., 2016).

## O-Fucosylation, C-Mannosylation, etc.

Epidermal growth factor (EGF) and thrombospondin repeat (TSR) domains on proteins can be glycosylated by O-Fuc, O-Glc, O-GlcNAc, and C-Man in flies and mammals; however, there are fewer data regarding the structures and importance of such modifications in *C. elegans*. Nevertheless, three relevant glycosyltransferases have been characterized: specifically the peptide-modifying O-fucosyltransferases POFUT1 and POFUT2 (both crystallized; the latter encoded by the *pad-2* gene) and tryptophan-modifying C-mannosyltransferase encoded by the *dpy-19* gene (Menzel et al., 2004; Lira-Navarrete et al., 2011; Buettner et al., 2013). There is an ortholog of the EGF-repeat-modifying EOGT *N-*acetylglucosaminyltransferase (Ogawa et al., 2015), but no *C. elegans* homolog of the Rumi protein required in other species for *O*-glucosylation of EGF repeats. Thus, it is probable that EGF and TSR domains in *C. elegans* are only modified by O-Fuc, O-GlcNAc and C-Man with no evidence yet that the O-Fuc will be elongated (Figure 4).

Quite well studied in *C. elegans* is cytosolic glycosylation with O-linked GlcNAc in which OGT-1 transfers, and OGA-1 removes, the monosaccharide, whereby OGT-1 is not related by sequence to the aforementioned EOGT (Hanover et al., 2005; Forsythe et al., 2006). On the other hand, while uncharacterized orthologs of the cadherin-modifying TMTC-type O-mannosyltransferases are encoded by the worm genome (Larsen et al., 2017), O-mannosylation of α-dystroglycan is probably absent due to a lack of homologs of the relevant POMT-type enzymes known in many other eukaryotes. A final type of O-glycosylation is a disaccharide modification of hydroxylysine residues on collagen and the *C. elegans* LET-268 protein has been reported to possess at least the relevant lysyl hydroxylase and galactosylhydroxylysyl glucosyltransferase activities (Wang et al., 2002).

## Glycosphingolipids and Glycolipid Anchors

Ceramide-based glycolipids are widespread in Nature and in many invertebrates they are based on Man β1,4Glc β1Cer (mactosyl ceramide or an *arthro* core, which contrast with the other glucosyl- or galactosylceramide-based structures in mammals). A variety of neutral and zwitterionic glycolipids have been described in *C. elegans* (see Figure 4), which are altered in the so-called *bre* mutants resistant to a *Bacillus* crystal toxin (Gerdt et al., 1997, 1999; Griffitts et al., 2005). Some relevant enzymes required for glycolipid biosynthesis (the CGT glucosyltransferases, the BRE-1/GMD-1 GDP-mannose dehydratase and an *N*-acetylgalactosaminyltransferase BRE-4) have been characterized *in vitro* (Kawar et al., 2002; Rhomberg et al., 2006; Nomura et al., 2011). Other classes of glycolipids include phosphoethanolamine glucosylceramides and the ascarosides, which both have signaling functions (Boland et al., 2017; von Reuss, 2018).

In terms of other glycolipid-like molecules, glycosylphosphatidylinositol (GPI) anchors on the C-termini of selected proteins, including some proteoglycans, occur in most eukaryotes and confer special properties in terms of protein trafficking and cell surface signaling. Although no structures of GPI anchors from *C. elegans* (or probably from any invertebrate animal) are known, 24 genes are predicted to encode proteins with roles in GPI biosynthesis and knock-out/down of some of these results in developmental phenotypes (Murata et al., 2012).

## Differences and Similarities Between *C. elegans* and Other Nematodes

**Figure 5:**
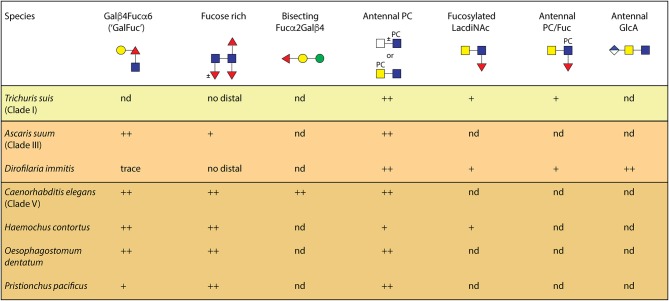
A comparison of N-glycan epitopes in different nematodes. This summary is based on our own work using an off-line HPLC-MALDI-TOF-MS workflow and shows the proven occurrence of various core and antennal modifications in nematodes. Additionally, whereas one methylated core α1,3-fucosylated glycan was detected in *P. pacificus* (Yan et al., 2015c), methylated or α-galactosylated fucose and mannose residues are present in *C. elegans* and *O. dentatum* (Yan et al., 2015b; Jiménez-Castells et al., 2017); glucuronylated N-glycans in nematodes have been only found thus far in *D. immitis* (Martini et al., 2019). The clades are those as defined by Blaxter (2011); “nd” signifies not detected to date, while ++/+ is an indication of the level of expression of the relevant epitope.

The structural diversity of N-glycans in *C. elegans* is very high, but there are differences in its glycosylation as compared to other nematodes, including parasitic species (summarized in Figure 5). The primary difference appears to be that no other nematode has four or five fucose residues associated with the core region. Maximally three have been detected on the N-glycan cores of *Oesophagostomum dentatum, Pristionchus pacificus* and *Haemonchus contortus*, and only two on the core of *Trichuris suis* (Haslam et al., 1996; Paschinger and Wilson, 2015; Yan et al., 2015c; Sutov, 2016; Wilson and Paschinger, 2016; Jiménez-Castells et al., 2017); galactosylation of the core α1,6-fucose is also a feature present in some nematode species including *Ascaris suum* and *Haemonchus contortus* (Yan et al., 2012; Paschinger and Wilson, 2015), but not in others such as *T. suis*. Methylation is also a recurrent, but not universal, modification of nematode N-glycans.

Antennal modifications such as *N-*acetylgalactosamine, Lewis-like fucose, chito-oligomer or even glucuronylated extensions, yet to be detected in *C. elegans*, have been found in, e.g., *H. contortus, T. suis, Dictyocaulus viviparous, O. dentatum, Onchocera volvulus*, and *Dirofilaria immitis* (Haslam et al., 1999, 2000; Paschinger and Wilson, 2015; Sutov, 2016; Wilson and Paschinger, 2016; Martini et al., 2019), whereas phosphorylcholine (PC) modifications are a common theme throughout the Nematoda, although the exact structures differ. For instance, *Trichinella spiralis* and *Acanthocheilonema viteae* synthesize PC-modified glycans with up to four antennae (Haslam et al., 1999; Morelle et al., 2000), whereas *C. elegans* and its closest relatives have maximally three, which reflects the different number of branching GlcNAc transferases encoded by their genomes.

As noted above, the hydrazine-released glycosaminoglycan-like oligosaccharides from *C. elegans* differ from the PC-modified ones found in *O. dentatum* (Vanbeselaere et al., 2018). Other comparisons of mucin-type O-glycans are difficult to make as the data is fragmentary, but currently it can be concluded that their structures do differ between nematode species (Khoo et al., 1991; Hewitson et al., 2016; Sutov, 2016). On the other hand, at least the modification of glycolipids by phosphorylcholine is shared between *C. elegans, Ascaris suum* and *Onchocera volvulus* (Lochnit et al., 1998; Wuhrer et al., 2000).

## Glycoanalytical Challenges

To date, as for most organisms, the vast majority of glycomic analyses on *C. elegans* wild-type or mutant strains have been on the N-glycans. This reflects that their release and isolation of is eased by the use of enzymes which can cleave most N-glycans under mild conditions: i.e., PNGase F and A. However, as noted above, some structures with highly modified cores (i.e., with three galactosylated fucose residues) can only be isolated after use of a special PNGase (the new PNGase Ar) or by hydrazinolysis (Yan et al., 2018a), a chemical method with some problems associated with safe handling and artifacts. Probably steric hindrance limits the access of standard PNGases to the GlcNAc-Asn bond if there are too many core modifications and, therefore, comparisons with other organisms now need to take account of means for release of such structures; certainly, two rounds of PNGase release are to be recommended even if using PNGase Ar.

Nevertheless, in terms of analyses, the N-glycome of *C. elegans* is not yet fully characterized, but key to its resolution continues to be an adequate LC-MS approach in which glycans from wild-type and mutant strains can be individually chromatographically resolved and then characterized by specific chemical and enzymatic treatments in combination with MALDI-TOF-MS/MS and “targeted” ESI-MS^n^ or, if amounts allow, NMR (Yan et al., 2015a). The advantage of pyridylamination (PA) as a fluorescent labeling method is the ability to easily detect the Y-fragments associated with reducing terminal core modifications and to separate structural isomers on HPLC (Hykollari et al., 2017); thereby, antennal and core fucosylation can be easily distinguished by MS/MS if the core is labeled and, if necessary, the glycans can be refractionated by HPLC after treatments with, e.g., α-mannosidases, α- or β-galactosidases, α-fucosidases, β-*N-*acetylhexosaminidases or hydrofluoric acid, whereas the numbers of isomeric structures, which would complicate any “all-at-once” analysis, are not to be underestimated. For instance, there were seven forms of Hex_6_HexNAc_2_ in the triple *gly-12;gly-13;gly-14* mutant and 13 isomers of Hex_4_HexNAc_2_Fuc_1_ when considering all double *fut* deletion strains; these structures could only be resolved by 2D-HPLC prior to MS/MS before and after enzymatic digestions which demonstrated varying numbers and positions of mannose and galactose residues (Yan et al., 2015b, 2018b). There is one disadvantage of pyridylamination and that is the lack of a free amino group for printing glycans in an array format; an alternative is 2-amino-*N*-(2-amino-ethyl)-benzamide (AEAB) as already used for *C. elegans* and *D. immitis* arrays (Jankowska et al., 2018; Martini et al., 2019), but this label seems to have poorer HPLC resolution and MS ionization properties.

On the other hand, permethylation as part of an analysis of intact glycans must be replaced by perdeuteromethylation to avoid missing the naturally methylated structures which do occur in nematodes (Haslam et al., 2002; Wohlschlager et al., 2014); the subsequent work-up with organic extraction leads to loss of zwitterionic-modified forms and their “backbone” structures are then only observed after hydrofluoric acid treatment to remove the phosphodiesters (Haslam et al., 1997). It will be interesting to see whether employing methods for sulpho-glycomics (Kumagai et al., 2013), based on solid phase extraction of the aqueous phase post-permethylation, can be adapted for PC-glycomics of released glycan chains. Alternatives for derivatising PC-modified glycans are perdeuteroacetylation (which modifies all hydroxyl residues) or the aforementioned pyridylamination (modifying just the reducing terminus), whereby using the latter label we have analyzed glycans with up to three or four PC moieties in nematodes other than *C. elegans* (Yan et al., 2015c; Jiménez-Castells et al., 2017). GC-MS linkage analyses using partially methylated alditol acetates did offer, e.g., a first clue as to the presence of a bisecting residue (now known to be galactose) and aided definition of the positions of galactose substitutions of core fucose modifications (Haslam et al., 2002; Yan et al., 2012); however, like NMR, for GC-MS larger amounts of pure material are required if wishing to analyse a single structure.

O-glycans, glycosaminoglycans, glycolipids, or GPI anchors present their own specific difficulties, as chemical or organic extraction methods are required for isolation. As O-glycans are differently modified or based on linkages to peptide with, e.g., O-GalNAc, O-Fuc, or O-Man, there is no single enzymatic method for their release. Although a number of non-reductive chemical approaches for O-glycan release have been described, β-elimination in the presence of borohydride is still the most widely used, but is incompatible with later fluorescent labeling via reductive amination of the reducing terminus; also, the small size of some O-glycans is a complication when trying to analyse real glycan signals as opposed to “dirt.” Hydrazinolysis can, though, be used for O-glycomics as well as for release of glycosaminoglycan chains (Vanbeselaere et al., 2018), but toxicity, artifactual peeling, partial demethylation of PC and “junk” peaks are negative aspects of its use.

Glycolipid analysis requires a different initial work-up as compared to protein-linked glycans and requires various multiple steps including chemical treatment (saponification) with potential effects on labile glycoconjugates or organic extraction/chromatography; with chloroform; however, use of an endoglycoceramidase can be followed by pyridylamination for analysis of the glycan moieties of nematode glycolipids (Gerdt et al., 1997, 1999). Recently, hypochlorite has been described as a method for isolating the glycan moieties of glycolipids (Song et al., 2016), but we are not aware of this having been applied to nematode samples. Finally, analyses of GPI anchors has only been performed in a few labs and seemingly not yet on nematodes, although at least two *C. elegans* lipid raft proteins have been confirmed experimentally to be releasable with the PI-PLC phospholipase (Rao et al., 2011).

## Nucleotide Sugar Metabolism

The biosynthesis of glycoconjugates requires activated sugars; also, other than for the first few reactions in N-glycan biosynthesis, these must then be transported across the endoplasmic reticulum and Golgi membranes in order to be utilized by the glycosyltransferases in the lumen of these organelles. Based on the composition of the different proven glycan structures, it is no surprise that *C. elegans* has the capacity to generate a number of nucleotide sugars such as GDP-Man, GDP-Fuc, UDP-Gal, UDP-Glc, UDP-GalNAc, UDP-GlcNAc, UDP-GlcA, and UDP-Xyl; some relevant NDP-sugar synthases from this organism have been characterized by enzymatic or genetic means (Hwang and Horvitz, 2002a,b; Rhomberg et al., 2006; Brokate-Llanos et al., 2014). However, it is not clear why this worm can synthesize UDP-Gal_*f*_ or dTDP-Rha as no galactofuranose- or rhamnose-containing glycoconjugates have yet been reported, but the UDP-Gal mutase gene is essential (Novelli et al., 2009) and expression of the rhamnose biosynthetic genes may be coupled to molting (Feng et al., 2016). In terms of transporters, which are actually antiporters with nucleoside monophosphates as counter-substrates, a number have been identified including ones accepting UDP-Gal, UDP-GalNAc, UDP-Glc, UDP-GlcNAc, UDP-GlcA, GDP-Fuc, and 3′-phospho-adenosine-5′-phosphosulfate (PAPS, the substrate for sulphotransferases) as cargo (Berninsone et al., 2001; Lühn et al., 2001; Höflich et al., 2004; Caffaro et al., 2008; Bhattacharya et al., 2009).

## Endogenous and Exogenous Lectins

Glycans make no biological sense unless recognized and so organisms need a range of carbohydrate-binding proteins. *C. elegans* is no exception and expresses a range of C-type lectins as well as galectins with either roles in innate immunity or endogenous physiology. These are respectively encoded by the *clec* and *lec* gene families; however, the specificity of the vast majority of C-type lectins, other than CLEC-79, has not been defined and commonly only sequence predictions or transcriptomics have been performed (Drickamer and Dodd, 1999; Schulenburg et al., 2008; Takeuchi et al., 2008). On the other hand, *C. elegans* has become an interesting model for testing the toxicity of lectins isolated from, e.g., fungi which defend themselves against soil-living nematodes (Bleuler-Martínez et al., 2011). Thereby, by feeding recombinant forms of these lectins, *in vivo* targets have been determined on the basis of the resistance of certain *C. elegans* glycomutant strains (Butschi et al., 2010; Schubert et al., 2012; Wohlschlager et al., 2014); however, as *C. elegans* is a bacteriophore, the probable major “battleground” for its survival *in vivo* will be against bacteria rather than fungi. Nevertheless, one of these fungal lectins, CGL2, binds the same epitope (“GalFuc”) as some of the worm's own galectins (Takeuchi et al., 2009; Maduzia et al., 2010; Nemoto-Sasaki et al., 2011), while *C. elegans* LEC-8 binds the worm glycolipids perhaps similarly to the Cry5B crystal toxin (Ideo et al., 2009). Certainly, a thorough evaluation of lectin binding to worm epitopes will require a well-defined glycan array.

## Conclusion

Twenty years ago, a review article was entitled “*Caenorhabditis elegans* is a nematode” (Blaxter, 1998), which of course is still true. However, in terms of N-glycans *C. elegans* is not necessarily a typical nematode; just as *Drosophila* is “the” model insect, but probably lacks some N-glycomic features of the Lepidoptera and Hymenoptera including zwitterionic modifications (Stanton et al., 2017; Hykollari et al., 2018), the glycome of *C. elegans* is far from identical to those of other nematodes. Although the trifucosylation of the core region, galactosylation of α1,6-fucose and phosphorylcholination are shared with many parasitic worms, the bisecting galactose is seemingly unique, while other features such as chito-oligomers or glucuronylated antennae may be absent from the model worm. Thus, only in part can we use *C. elegans* as a surrogate to understand aspects of the roles of glycans in, e.g., host-parasite interactions and some peculiarities of the biosynthesis or structure of its glycans make it quite distinct from any mammalian model. For the preparation of glycan arrays or the isolation of some specific glycosyltransferase genes we must still rely on organisms whose life cycles depend on animal or plant hosts in order to extend our knowledge about nematode glycosylation. There is indeed a long way to go and some aspects, such as plant parasites, have been completely neglected in glycobiological terms. Therefore, despite some 20 years of work on the N-glycomes of nematodes, there is much exciting and challenging work remaining to be done.

## Author Contributions

KP, SY, and IW wrote and reviewed the manuscript.

### Conflict of Interest Statement

The authors declare that the research was conducted in the absence of any commercial or financial relationships that could be construed as a potential conflict of interest. The handling editor and reviewer MAW declared their involvement as co-editors in the Research Topic, and confirm the absence of any other collaboration.
